# Use of healthcare services before diagnosis of attention-deficit/hyperactivity disorder: a population-based matched case-control study

**DOI:** 10.1136/archdischild-2023-325637

**Published:** 2023-10-30

**Authors:** Vibhore Prasad, Emma Rezel-Potts, Patrick White, Johnny Downs, Nicholas Boddy, Kapil Sayal, Edmund Sonuga-Barke

**Affiliations:** 1 Population Health Science, King's College London, London, UK; 2 Institute of Psychiatry, Psychology, and Neuroscience, King’s College London, London, UK; 3 School of Medicine, University of Nottingham, Nottingham, Nottinghamshire, UK; 4 CANDAL (Centre for ADHD and Neuro-Developmental Disorders Across the Lifespan), Institute of Mental Health, University of Nottingham, Nottingham, UK

**Keywords:** child health, adolescent health, child psychiatry, primary health care, epidemiology

## Abstract

**Objective:**

To compare use of healthcare services and reasons for attendance by children and young people (CYP) with attention-deficit/hyperactivity disorder (ADHD) versus non-ADHD controls.

**Design:**

Population-based matched case-control study.

**Setting:**

English primary care electronic health records with linked hospital records from the Clinical Practice Research Datalink, 1998–2015.

**Participants:**

8127 CYP with an ADHD diagnosis aged 4–17 years at the time of diagnosis and 40 136 non-ADHD controls matched by age, sex and general practitioner (GP) practice.

**Main outcome measures:**

Medical diagnoses, prescriptions, hospital admissions and hospital procedures in the 2 years before diagnosis (or the index date for controls).

**Results:**

CYP with ADHD attended healthcare services twice as often as controls (rate ratios: GP: 2.0, 95% CI=2.0, 2.1; hospital 1.8, 95% CI=1.8, 1.9). CYP with ADHD attended their GP, received prescriptions and were admitted to hospital for a wide range of reasons. The strongest association for GP attendances, comparing CYP with versus without ADHD, was for ‘mental and behavioural disorders’ (OR=25.2, 95% CI=23.3, 27.2). Common reasons for GP attendance included eye, ear, nose, throat, oral (OR=1.5, 95% CI=1.4, 1.5) and conditions such as asthma (OR=1.3, 95% CI=1.3, 1.4) or eczema (OR=1.2, 95% CI=1.0, 1.3).

**Conclusions:**

Two years before diagnosis, CYP with ADHD attended healthcare services twice as often as CYP without. CYP with ADHD had increased rates of physical conditions, such as asthma and eczema. These contacts may be an opportunity for earlier recognition and diagnosis of ADHD.

**WHAT IS ALREADY KNOWN ON THIS TOPIC**

- Attention-deficit/hyperactivity disorder (ADHD) is the most frequent neurodevelopmental disorder among children and young people (CYP), with an estimated prevalence of 3%–5%.
- Diagnosis of ADHD in CYP is often delayed or missed and this may impede access to effective treatments.
- Previous work has suggested that general practitioners (GPs) have difficulty recognising ADHD.

**WHAT THIS STUDY ADDS**

- Compared with controls, CYP with ADHD have attended their GP and general hospital services twice as often in the 2 years preceding their diagnosis.
- CYP attend for a range of reasons, including mental and physical reasons unrelated to ADHD.
- These frequent contacts of CYP with a range of healthcare services may present an opportunity for earlier diagnosis.

**HOW THIS STUDY MIGHT AFFECT RESEARCH, PRACTICE OR POLICY**

- Further research is required to investigate the reasons for delayed diagnosis of ADHD.
- Parents/caregivers with concerns about ADHD should be encouraged to discuss these with the GP or specialist services.
- Care pathways should aim to be holistic so that concerns about physical, mental health and behavioural difficulties can be considered at the same time.
- Integrated care systems should be alert to serving the needs of CYP with symptoms of ADHD across health, education and social care services.

## Introduction

Attention-deficit/hyperactivity disorder (ADHD) is a prevalent neurodevelopmental disorder affecting 3%–5% of children and young people (CYP)^1^ characterised by developmentally inappropriate hyperactivity, impulsivity and inattention in multiple settings (eg, school and home).^2^ Peak age for diagnosis of ADHD is at 7–9 years.^3^ Yet, as a neurodevelopmental disorder, ADHD is likely to have been present from an earlier age, with persistent symptoms.^2 4^ ADHD is under-recognised, with <1% of CYP having a diagnosis in medical records,^1 5^ often with considerable delays, which are not well understood.^6^ Girls are disproportionately affected by underdiagnosis and delays in diagnosis,^7^ which results in transitioning from primary/junior school (4–11 years) to senior school (12–17 years) with inadequate support. ADHD is associated with poor mental health and negative outcomes across the life course,^8–12^ such as poor relationships and social functioning, low self-esteem,^12^ academic under-attainment^13–15^ and injuries.^16^

Outcomes can be improved by specialist services and evidence-based interventions,^1 2^ but access to these is often significantly delayed.^6^ One reason for this is that general practitioners (GPs), who are the main gatekeepers to specialist services in the UK,^1 2^ have difficulty recognising ADHD,^17 18^ leading to delay in referral. Identifying early markers of and wider health presentations associated with undiagnosed ADHD represents an opportunity to improve recognition and outcomes. In this study, we investigate the pattern of healthcare utilisation of CYP with ADHD in the 2 years before diagnosis to explore opportunities for earlier recognition and referral.

## Methods

### Data source

The Clinical Practice Research Datalink (CPRD) is a primary care database containing records of 15.2 million people from 730 GP practices and covers 7% of the UK population.^19 20^ We conducted a case-control study using medical records from just over half of CPRD practices in England who consented to linked hospital medical records from the Hospital Episodes Statistics (HES) database. HES contains the diagnoses associated with all overnight National Health Service (NHS) admissions and operative procedures.^21^ Previous work including all ages has shown a high validity of CPRD diagnoses, with a median of 89% of cases confirmed by manual review of GP records.^22^ The HES-linked CPRD is representative of the UK population in terms of age and sex, covering practices from every Strategic Health Authority (SHA) region of England.^23 24^ The CPRD contains Read (diagnostic and attendance) codes and drug codes arising from attendances at GPs. It also contains Read-coded correspondence from attendances at secondary care at outpatients, emergency departments, overnight admissions or operative procedures. CPRD contains Index of Multiple Deprivation (IMD) 2015, based on home postcode as a proxy measure of an individual’s socioeconomic status.^25^ HES data are coded using International Classification of Disease, V.10 (ICD10)^26^ and procedure codes from the Office of Population Censuses and Surveys, V.4 (OPCS4).^27^

### Study population

The study period was 1 January 1998 (HES-link started) to 31 December 2015 (data extraction). Individuals were eligible for inclusion if (1) they were 4–17 years of age during the study period, (2) they had been registered for at least 2 years in a primary care practice, which met research data recording standards (known as ‘up to standard’ and defined using CPRD algorithms examining patterns of data completeness and temporal gaps in recording), (3) they had at least 2 years of medical records prior to the index date. The index date was defined as the date of the first code for ADHD in the matched ADHD patient record. Events in the first 90 days included of medical records were ineligible because historical diagnoses may be incorrectly recorded as new diagnoses in the first few months of registering with a new GP practice.^28^

Cases had at least one drug/diagnostic code for ADHD (see online supplemental tables 1 and 2 for list of codes).

10.1136/archdischild-2023-325637.supp1 Supplementary data

Controls had no record of ADHD in their GP medical records. Up to five controls were randomly selected and matched on age (year of birth), sex, practice and index date.

### Reasons for attendance at healthcare services

The medical records from the CPRD GOLD and linked databases were extracted for participants in the 2 years before the index date.^19^ All codes were manually reviewed and categorised based on existing classification systems (ICD10,^26^ British National Formulary chapter headings^29^ and OPCS4 chapter headings)^27^ and clinical review (PW and VP).

### Analysis

#### Comparison of reasons for attendance by category between cases and controls

The number of people with one or more attendance in the 2 years prior to index date was counted according to category for: (1) Read codes (GP attendance and hospital correspondence); (2) drug codes (GP prescriptions); (3) ICD10 (hospital admissions); and (4) OPCS4 codes (hospital procedures). This number was the numerator. For example, a CYP who attended the GP more than once for a respiratory condition would contribute once to the analysis for respiratory Read codes. The denominator was the total number of cases in the study population. The outcome was presented as the proportion of attendances among the case and controls. Conditional logistic regression compared CYP cases to controls. We calculated relative risk estimates as ORs with 95% CIs. Where there were less than 20 cases or controls, the category was omitted from the results for clarity and to protect anonymity.

### Reasons for attendance

The three most frequently occurring codes resulting from CYP’s healthcare attendances were inspected for each type of code (online supplemental tables 4–7). We compiled a list of physical and mental health conditions for which CYP with ADHD might attend their GP (online supplemental table 3). To identify these conditions, we compiled a list of Read and drug codes (VP and PW). We reviewed the medical records of CYP with versus without ADHD for at least one attendance with the condition. An unadjusted OR was estimated for the condition using conditional logistic regression.

To explore the frequency of healthcare service use between cases and controls, the attendance rates were described for CYP with versus without ADHD separately for Read codes, drug codes, ICD10 codes, OPCS4 codes. The frequency was described for GP attendances versus hospital admissions.

To assess whether patterns of healthcare attendance varied by sex or age, the proportions with each category of attendance were examined separately by sex and age (<11 years vs 12 years+, reflecting later diagnosis of ADHD). Age bands were chosen to reflect UK schooling (infant, junior and secondary) ages. We undertook subgroup analyses assessing the effect on our findings through varying the definition of an ADHD diagnosis as (1) at least two drug codes and at least two diagnosis codes, (2) at least two diagnosis codes and less than two drug codes, (3) at least two drug codes and less than two diagnosis codes and (4) one drug code or one diagnosis code/one drug code and one diagnosis code. Descriptive analyses are reported because of the size of the dataset and the large number of comparisons.

Lists of all Read and drug codes are available from the authors on request. Statistical analysis was performed using Stata V.15.1 (StataCorp, College Station, Texas, USA).

## Results

Table 1 shows the sample characteristics (8127 cases and 40 136 controls).

**Table 1:** Characteristics of children and young people with and without ADHD

Characteristic	Cases n=8127	Controls n=40 136
Sex, n (%)		
Male	6716 (82.6)	33 164 (82.6)
Age group at diagnosis (years), n (%)		
4	176 (2.2)	1013 (2.5)
5–6	1445 (17.8)	7092 (17.7)
7–10	3661 (45.1)	17 943 (44.7)
11–17	2845 (35.0)	14 088 (35.1)
Period of start of study, n (%)		
1998–1999	2137 (26.3)	11 185 (27.9)
2000–2004	3548 (43.6)	17 373 (43.3)
2005–2009	2062 (25.4)	9965 (24.8)
2010–2015	380 (4.7)	1613 (4.0)
Deprivation quintile, n (%)		
1st quintile (least deprived)	1355 (16.7)	8879 (22.1)
2nd quintile	1366 (16.8)	7818 (19.5)
3rd quintile	1527 (18.8)	7769 (19.4)
4th quintile	1751 (21.6)	7602 (18.9)
5th quintile (most deprived)	2120 (26.1)	8020 (20.0)
Data missing	8 (0.1)	48 (0.1)
Region, n (%)		
North East	193 (2.4)	958 (2.4)
North West	1118 (13.8)	5517 (13.8)
Yorkshire & The Humber	164 (2.0)	813 (2.0)
East Midlands	263 (3.2)	1289 (3.2)
West Midlands	763 (9.4)	3757 (9.4)
East of England	1180 (14.5)	5789 (14.4)
South West	787 (9.7)	3887 (9.7)
South Central	1206 (14.8)	5974 (14.9)
London	871 (10.7)	4297 (10.7)
South East Coast	1582 (19.5)	7855 (19.6)

Sex, age and practice were matching variables. Therefore, cases and controls were registered at the same GP practice.

ADHD, attention-deficit/hyperactivity disorder ; GP, general practitioner.

CYP with ADHD were twice as likely as controls to have contact with the health services, prior to diagnosis, regardless of the type of contact (GP consultation, prescription, hospital admission and procedure) (GP: 8 vs 4; hospital 0.2 vs 0.1, times per year) (table 2).

**Table 2:** Rates for contact with health service by type of contact (GP consultation, prescription, hospital admission and procedure) for children and young people with vs without ADHD (n=48 263)

Type of health service contact	With ADHD Contacts per year n=8127	95%CI	Without ADHD Contacts per year n=40 136	95% CI	Rate ratio	95% CI
GP						
Diagnoses	6.90	6.86 to 6.94	3.24	3.23 to 3.26	2.13	2.12 to 2.14
Prescriptions	2.56	2.54 to 2.59	1.74	1.73 to 1.75	1.47	1.46 to 1.48
Diagnoses or prescriptions	8.01	7.96 to 8.05	3.92	3.91 to 3.94	2.04	2.03 to 2.05
Hospital						
Diagnoses	0.17	0.16 to 0.17	0.09	0.09 to 0.09	1.84	1.80 to 1.88
Procedures	0.09	0.09 to 0.09	0.06	0.06 to 0.06	1.54	1.48 to 1.60
Diagnoses or procedures	0.20	0.19 to 0.21	0.11	0.11 to 0.11	1.82	1.78 to 1.86

Average yearly rate over two years of follow-up.

ADHD, attention-deficit/hyperactivity disorder; GP, general practitioner.

**Figure 1:**
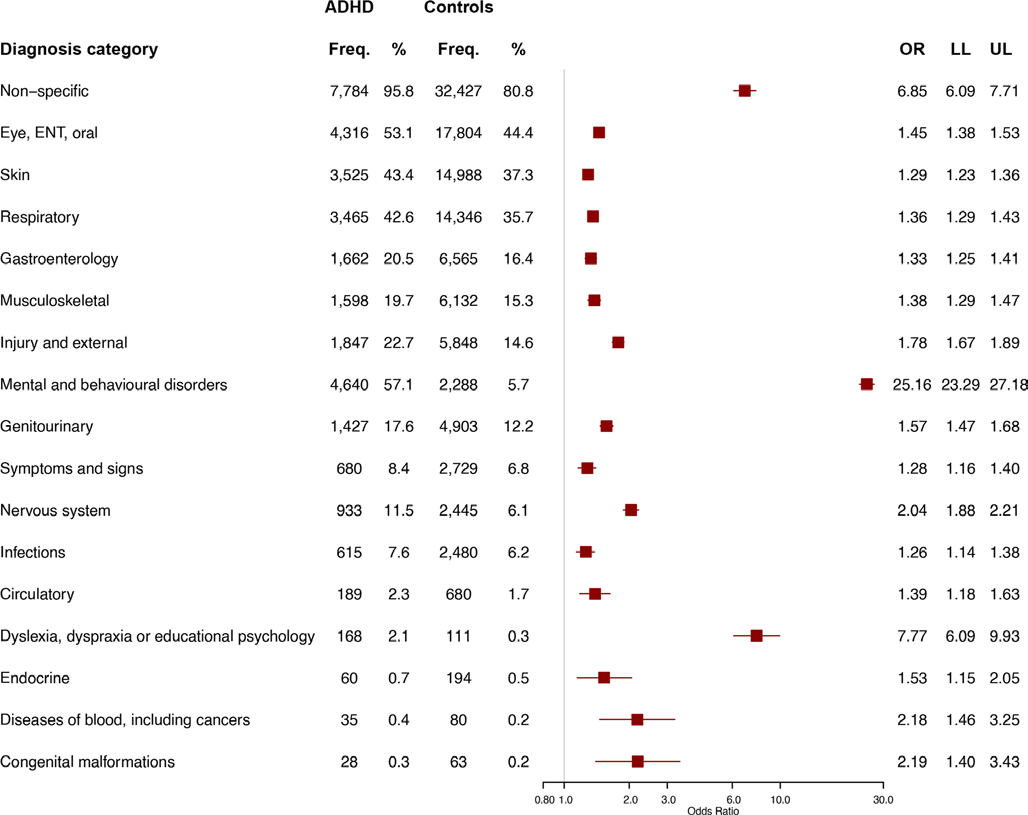
Attendances to the GP by categories of diagnoses (Read codes), comparing children and young people with (n=8127) versus without (n=40 136) ADHD (n=48 263). Categories are arranged in order of frequency for CYP with ADHD. The square shows the OR and the horizontal line shows the 95% CI. ADHD, attention-deficit/hyperactivity disorder; CYP, children and young people; Freq., frequency; GP, general practitioner; LL, lower limit of the 95% CI; UL, upper limit of the 95% CI.

**Figure 2:**
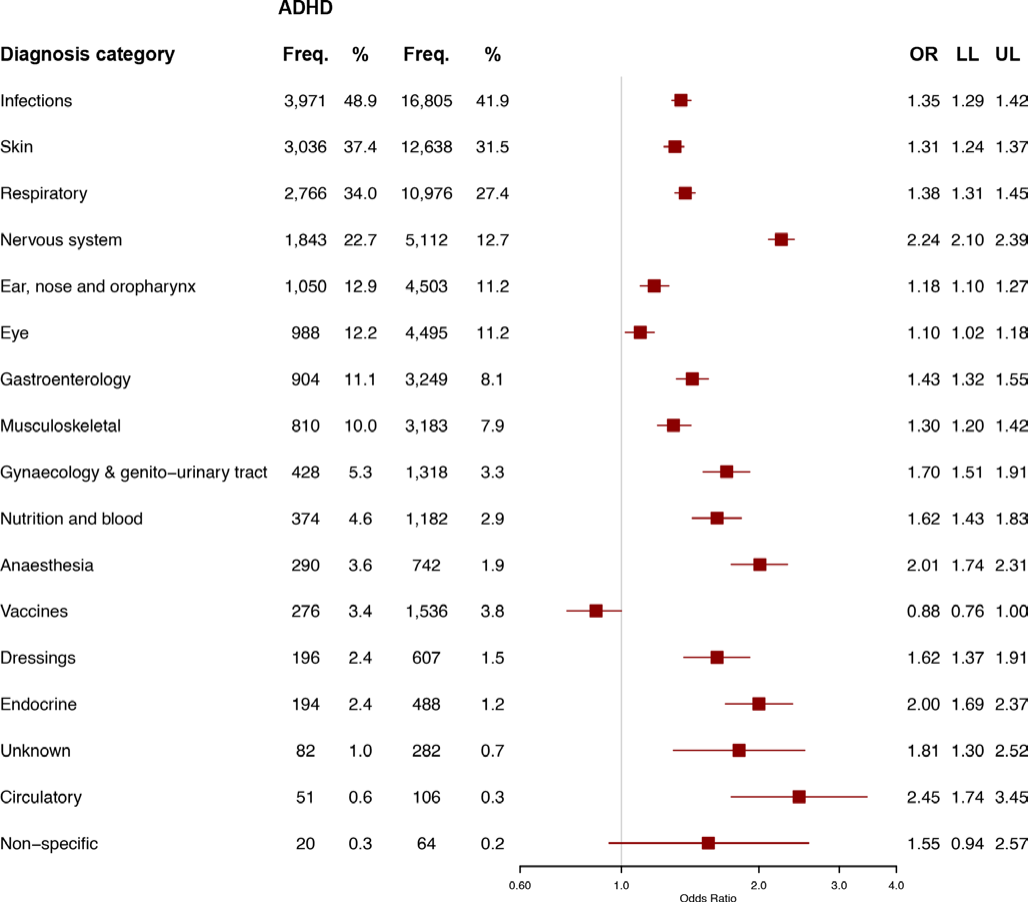
Attendances to the GP by categories of drugs (drug codes), comparing children and young people with (n=8127) versus without (n=40 136) ADHD (n=48 263). Categories are arranged in order of frequency for CYP with ADHD. The square shows the OR and the horizontal line shows the 95% CI. ADHD, attention-deficit/hyperactivity disorder; CYP, children and young people; Freq., frequency; GP, general practitioner; LL, lower limit of the 95% CI; UL, upper limit of the 95% CI.

The odds of cases having attended their GP were greater than controls in all 17 categories (figure 1). The strongest association was for ‘mental and behavioural disorders’ (OR=25.2, 95% CI=23.3, 27.2). The odds for cases receiving a prescription were greater in 16 of 17 categories (figure 2). The strengths of the association with a ‘circulatory’ prescription (OR=2.5, 95% CI=1.7, 3.5) and a nervous system prescription (OR=2.2, 95% CI=2.1, 2.4) were similar but the former was an uncommon event.

**Figure 3:**
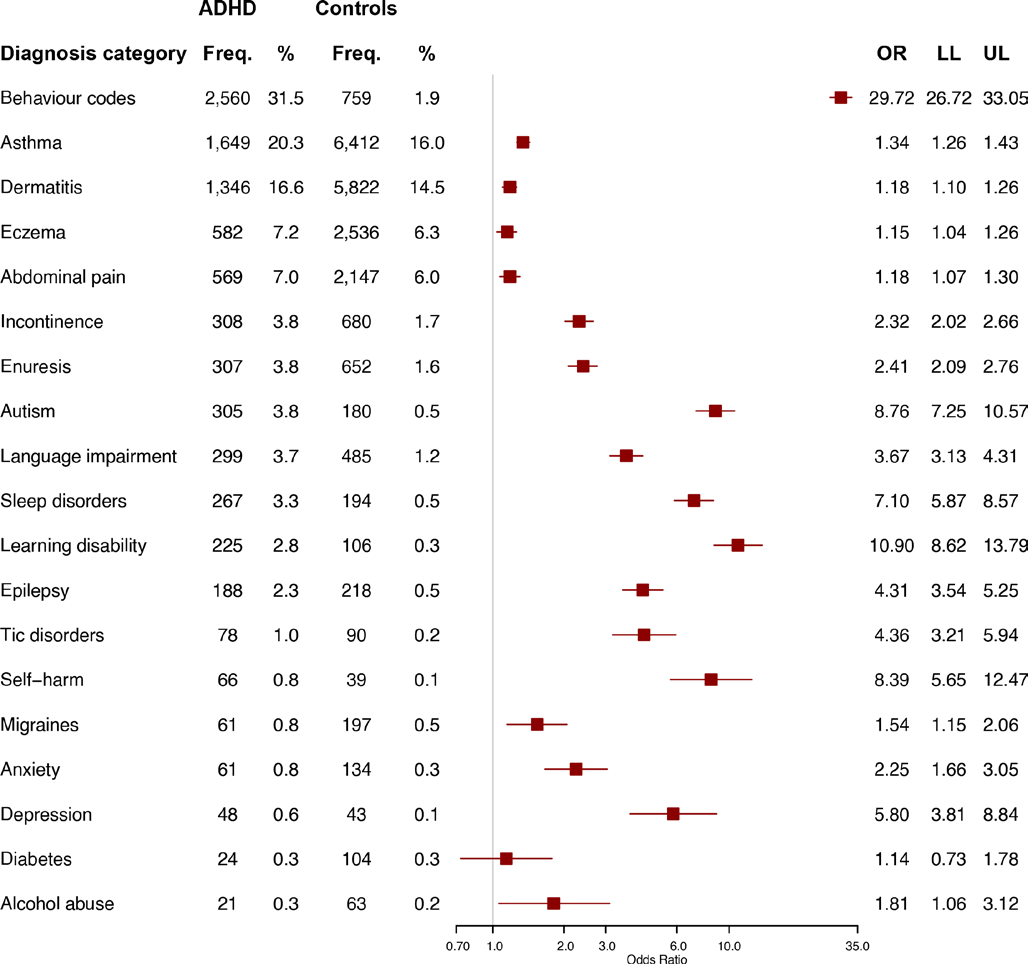
Medical conditions recorded in the GP medical records, comparing children and young people with (n=8127) versus without (n=40 136) ADHD (n=48 263). Categories are arranged in order of frequency for CYP with ADHD. ADHD, attention-deficit/hyperactivity disorder; CYP, children and young people; Freq., frequency; GP, general practitioner; LL, lower limit of the 95% CI; UL, upper limit of the 95% CI.

Figure 3 shows medical conditions recorded in the GP medical records in the 2 years before diagnosis. For 18 out of 19 conditions, the odds were greater for cases. The strongest associations were for ‘behaviour codes’ (OR=29.7, 95% CI=26.7, 33.1) and ‘learning disability’ (OR=10.9, 95% CI=8.6, 13.8).

**Figure 4:**
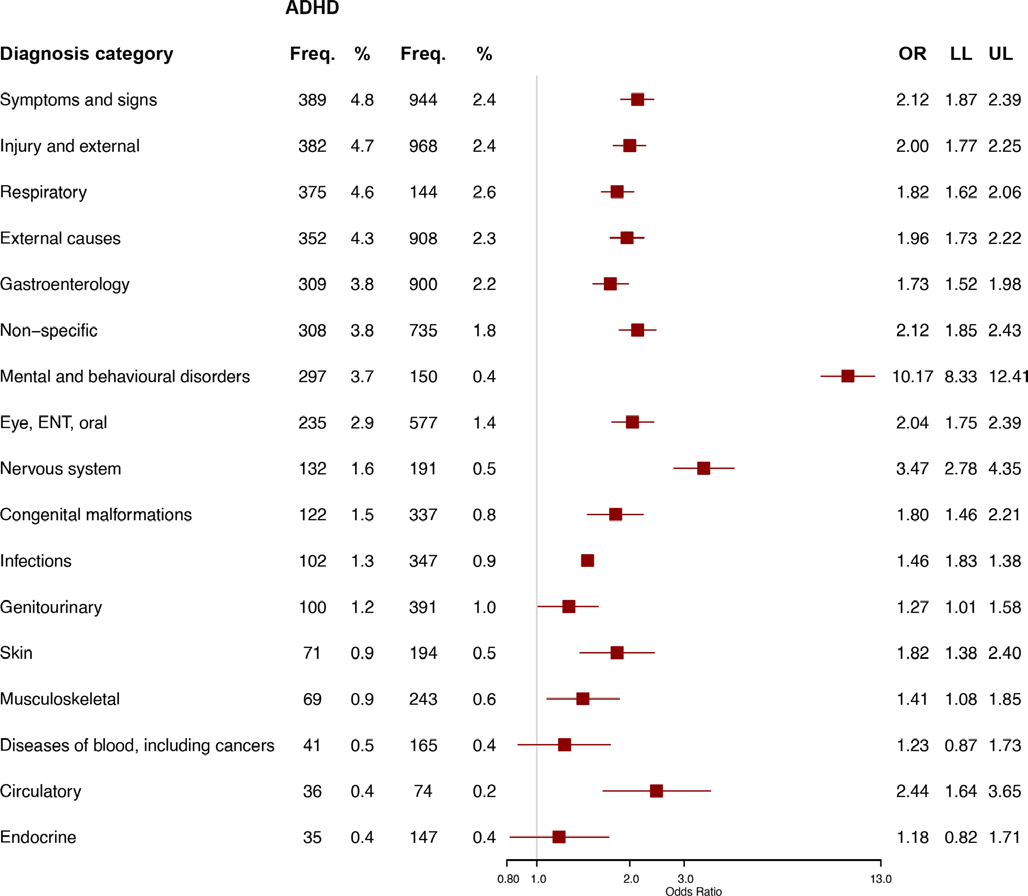
Admissions to hospital by categories of diagnoses (ICD10 codes), comparing children and young people with (n=8127) versus without (n=40 136) ADHD (n=48 263). Categories are arranged in order of frequency for CYP with ADHD. ADHD, attention-deficit/hyperactivity disorder; ENT, ear, nose and throat; Freq., frequency; LL, lower limit of the 95% CI; UL, upper limit of the 95% CI.

Figure 4 shows admissions to hospital in the 2 years before diagnosis. For 15 out of 17 categories, the odds were greater for cases. The strongest association was for ‘mental and behavioural disorders’ (OR=10.2, 95% CI=8.3, 12.4).

For 12 out of 13 categories, the odds were greater for cases for hospital procedures (online supplemental figure 1). Sex-stratified analyses showed little difference between males and females. However, there were exceptions (eg, compared with males, female cases had a stronger association for: GP attendances (musculoskeletal, injuries); prescriptions (nervous system); medical conditions (language impairment and autism); hospital (respiratory, injuries, nervous system and external causes) (online supplemental figures 2–6). Age-stratified analyses showed little difference between 4–11 and 12–17 years. However, there were exceptions (eg, 4–11 year old cases had a stronger association for GP attendances (respiratory, injuries, infections); prescriptions (nervous system, eye); medical conditions (tics, abdominal pain, behaviour, self-harm); hospital (injuries, external causes); hospital procedures (bones and joints) (online supplemental figures 7–11). Varying the definition of ADHD did not alter the results.

10.1136/archdischild-2023-325637.supp2 Supplementary data

## Discussion

### Main findings

In the 2 years preceding their diagnosis, CYP with ADHD used health services significantly more than controls. Their rates of attendance to GPs and hospitals were higher across almost all presentation categories investigated. The largest differences were seen in mental and neurodevelopmental presentations but there were increased rates of physical conditions, such as asthma and eczema. They also received treatment twice as often and were more likely to have records of other physical and mental health conditions. These findings suggest that there were potential opportunities for earlier recognition.

### Strengths and weaknesses

To our knowledge, this is the first work to investigate the primary and secondary healthcare attendances (using English NHS healthcare records) prior to diagnosis of ADHD. The CPRD-HES linked database provides the most detailed available picture of healthcare attendances. Previous work has shown that the CPRD-HES linked data are representative of GP and hospital attendances in the UK population and there is no reason to suspect findings from HES-linked practices differ from the overall CPRD.^5 16 22–24 30^ However, there were limitations. Although ADHD misdiagnosis is unlikely because it is diagnosed by specialists according to national guidelines,^2^ a small number (<1% of CYP) had a record of ADHD in their hospital but not GP records. This may result from delays in communication from hospital. There was a high proportion of attendances for ‘behavioural’ reasons which implies that GPs may have been aware that some presentations were suggestive of ADHD. GPs use Read codes for clinical recording and do not primarily collect these data for research. However, national guidelines^2^ specify referral to specialist services for assessment of ADHD, perhaps resulting in inherent delays to diagnosis, especially if there are long waiting lists.^6^ Presentations to healthcare may arise from parents/caregivers having a low threshold for seeking help when their child was unwell for other reasons and when they had other long-term medical conditions, resulting in ascertainment bias. However, there were a greater number of hospital admissions and procedures for CYP with ADHD which might imply more severe presentations, and not accounted for by lower help-seeking thresholds. It is also possible that the true association between health problems and ADHD may be smaller than our estimates. These data were extracted from the CPRD to cover the 1998–2015 period. Recent reviews have implied potential for overdiagnosis of ADHD,^1 31^ mainly in the USA and reported under-recognition in the UK CPRD up to 2010.^3 32^ However, since cases and controls were matched by age, it is unlikely that temporal trends in recognition (eg, rising recognition of ADHD) were different between cases and controls. Although we matched cases and controls on age, sex and practice, there may have been other confounders (such as parental mental health problems including parental ADHD) that we were unable to account for. Individual postal codes are unavailable from CPRD to protect anonymity. CYP attend healthcare for a wide range of reasons. Due to the nature of Read codes, which include codes for GP administration and symptoms, a high proportion of attendances were classified as ‘factors influencing health status/contact with services’. Although the reason for these attendances is unclear, it is unlikely that the effect would have been different between cases and controls.

### Comparison with existing literature

Our findings are consistent with previous studies suggesting that CYP with ADHD are at an increased risk of having other mental or physical health disorders, including injuries.^16^ For example, in a German health insurance database study, 83% with ADHD (vs 20% without) had a comorbid psychiatric diagnosis and 2% (vs 1.3%) had a cardiovascular disorder.^33^ A Korean health insurance database study reported a range of associations between ADHD and other disorders, for example, nervous system disease (OR=2.59), which was similar to our findings.^34^ However, the health events were based only on hospital attendances, which may not represent the full range of disorders or healthcare presentations. A global systematic review reported a similar association between ADHD and asthma as our findings.^35^ The male to female ratio was 4.8:1 in our sample which is similar to previous studies from healthcare data in the UK (5:1)^36^ and Germany (3-4:1).^37^ There is some suggestion that, compared with males, females with ADHD have more injuries, overnight admissions for respiratory conditions, receive more nervous system drugs, and have recorded autism and language impairments. These findings warrant further research and may address the unequal recognition of females with ADHD.^7^

### Implications for future research

CYP with ADHD had a higher risk of mental health or behaviour codes, dyspraxia, dyslexia, autism, tics and insomnia being recorded prior to diagnosis. These codes might suggest an opportunity for earlier diagnosis or may indicate clinicians were already gathering information (eg, related to other neurodevelopmental conditions), which would eventually lead to an ADHD diagnosis. These findings warrant further research. Further research is also required to develop and test interventions to identify ADHD earlier in primary care.^38^ For example, machine learning in CPRD data may generate a predictive model for automated detection of ADHD among patients with no formal diagnosis, such as those generated for other conditions^39^ or in other datasets.^40^ CYP with ADHD have multiple health needs and long-term vulnerabilities. Research exploring how CYP with ADHD might interface differently with health services might be useful in understanding the overlap of mental and physical health needs among CYP presenting in primary care, especially as CYP with ADHD have high healthcare costs.^32^ However, our work suggests that CYP with ADHD may also incur greater healthcare costs prior to their diagnosis.

### Implications for practice

While acknowledging that GPs may not recognise reasons for attendance as being related to ADHD^18^ and that parent/caregiver perceptions of a behaviour problem influence recognition,^18 41^ our work suggests there are potential earlier opportunities to identify undiagnosed ADHD. Healthcare practitioners (primary and secondary care), and Integrated care systems in health, education and social care services, should be aware that CYP who attend frequently and for a wide variety of reasons may have additional needs reflecting an unrecognised healthcare problem, such as ADHD.

## Data Availability

Data may be obtained from a third party and are not publicly available. The data that support the findings of this study are available by application to CPRD directly via www.cprd.com.
