# Supplementary material for: Use of healthcare services before diagnosis of attention-deficit/hyperactivity disorder: a population-based matched case-control study
Source: Arch Dis Child. 2023 Oct 30;109(1):46–51. doi: 10.1136/archdischild-2023-325637 (PMC10803994; doi:10.1136/archdischild-2023-325637)
Supplement: Supplementary data [file archdischild-2023-325637supp001.pdf]

Attention-deficit/hyperactivity disorder and healthcare service utilisation: a case control study: Supplementary tables

Supplementary Table 1 Descriptions for diagnosis codes for Attention-Deficit/Hyperactivity Disorder (ADHD)

**Code descriptions - ADHD**
ADD - Attention deficit disorder
Attention deficit disorder
Attention deficit with hyperactivity
Attention deficit without hyperactivity
Child attention deficit disorder
Child attention deficit disorder NOS
Childhood hyperkinetic syndrome
DAMP - Deficits in attention motor control and perception
Deficits in attention motor control and perception
Disorders of attention and motor control
Hyperkinetic syndrome NOS
[X]Attention deficit disorder
[X]Attention deficit disorder
[X]Attention deficit hyperactivity disorder
[X]Deficits in attention, motor control and perception
[X]Disturbance of activity and attention
[X]Hyperkinetic disorder, unspecified
[X]Hyperkinetic disorders
[X]Hyperkinetic reaction of childhood or adolescence NOS
[X]Hyperkinetic syndrome NOS
[X]Other hyperkinetic disorders

Supplementary Table 2 Descriptions for drug codes for ADHD

**Drugs identifying ADHD**
amfetamine/dexamfetamine sulphate
atomoxetine hydrochloride
dexamfetamine sulphate
methylphenidate hydrochloride
modafinil
pemoline

Supplementary Table 3 Physical and mental health conditions for which CYP with ADHD might attend their GP

**Condition name**
Abdominal pain
Alcohol abuse
Anxiety
Asthma
Autism
Behaviour codes
Bipolar disorder
Cardiovascular conditions
Depression
Dermatitis
Diabetes
Eczema
Enuresis
Epilepsy
Incontinence
Language impairment
Learning disability
Migraines
Obsessive compulsive disorder
Self-harm
Sleep disorder
Substance abuse
Tic disorder

Supplementary Table 4 Most commonly occurring first GP attendance Read codes within each category of diagnosis for children and young people with and without ADHD (n=48,263)

	Cases (*n*=8,127)		Controls (*n*=40,136)
**GP diagnoses (Read codes), *n***			
**Infectious and parasitic disease**		**Infectious and parasitic disease**	
Chickenpox-varicella (A52..00)	209	Chickenpox-varicella (A52..00)	859
Enterobiasis – threadworm (AC74.00)	119	Enterobiasis – threadworm (AC74.00)	380
Worms in the family (65PA.11)	38	Chickenpox (A52..00)	178
**Neoplasms, cancer and diseases of the blood**		**Neoplasms, cancer and diseases of the blood**	
Iron deficiency anaemias (D00..00)	21	Iron deficiency anaemias (D00..00)	40
[M] Gliomas (BBb..00)	2	Benign neoplasm of skin (B76..00)	7
-	-	Cancer care review (8BAV.00)	6
-	-	Dermatofibroma (B76..13)	6
**Endocrine**		**Endocrine**	
Seen in diabetic clinic (9N1Q.00)	14	Seen in diabetic clinic (9N1Q.00)	46
Precocious puberty (Clz1000)	5	Gynaecomastia (K311000)	18
Gynaecomastia (K311000)	4	Seen in endocrine clinic (9N1s.00)	16
-	-	Type 1 diabetes mellitus (C10E.00)	11
**Mental and behavioural disorders**		**Mental and behavioural disorders**	
[V] Behavioural problems (ZV40.11)	918	[V] Behavioural problems (ZV40.11)	272
Behaviour disorder (E2C..11)	487	Behaviour disorder (E2C..11)	131
Seen in psychiatry clinic (9N1T.00)	386	Seen in psychiatry clinic (9N1T.00)	96
**Diseases of the nervous system**		**Diseases of the nervous system**	
Seen in speech and language clinic (9N0Q.00)	188	Headache (1B1G.00)	561
C/O – a headache (1B1G.11)	123	C/O – a headache (1B1G.11)	513
Headache (1B1G.00)	117	Seen in speech and language clinic (9N0Q.00)	287
**Eye, ear, nose and throat, mouth and dental**		**Eye, ear, nose and throat, mouth and dental**	
Acute tonsillitis (H03..00)	373	Acute tonsillitis (H03..00)	1908
Sore throat symptom (1C9..00)	321	Sore throat symptom (1C9..00)	1463
Acute conjunctivitis (F4C0.00)	190	Acute conjunctivitis (F4C0.00)	854
**Circulatory system**		**Circulatory system**	
Seen in cardiac clinic (9N1P.00)	34	Seen in cardiac clinic (9N1P.00)	104
O/E – heart sounds normal (24B1..00)	9	Henoch-Schonlein purpura (D310000)	34
Palpitations (181..00)	8	Palpitations (181..00)	32
**Respiratory**		**Respiratory**	
C/O – cough (171..11)	477	C/O – cough (171..11)	2085
Cough (171..00)	450	Cough (171..00)	1809
Upper respiratory tract infection NOS (H05z.11)	414	Upper respiratory tract infection NOS (H05z.11)	1740
**Gastroenterology**		**Gastroenterology**	
Abdominal pain (16969.00)	237	Abdominal pain (16969.00)	1166
Vomiting (1992.00)	210	Vomiting (1992.00)	845
[D] Abdominal pain (R090.00)	118	[D] Abdominal pain (R090.00)	467
**Skin**		**Skin**	
C/O: a rash (1D14.00)	431	C/O: a rash (1D14.00)	1774
Impetigo (M05..00)	301	Impetigo (M05..00)	1049
Atopic dermatitis/eczema (M111.00)	175	Atopic dermatitis/eczema (M111.00)	869
**Musculoskeletal and connective tissue**		**Musculoskeletal and connective tissue**	
Seen in orthopaedic clinic (9N1p.00)	272	Seen in orthopaedic clinic (9N1p.00)	929
Knee pain (1M10.00)	92	Knee pain (1M10.00)	318
Seen in physiotherapy department (9N1yE00)	50	Cervicalgia (N131.00)	172

	Cases (*n*=8,127)		Controls (*n*=40,136)
**Genitourinary including gynaecology**		**Genitourinary including gynaecology**	
Enuresis (1A22.00)	134	Balanitis (K271.11)	432
Balanitis (K271.11)	125	Dysuria (1A55.00)	384
Dysuria (1A55.00)	86	Enuresis (1A22.00)	301
**Pregnancy, childbirth, puerperium**		**Pregnancy, childbirth, puerperium**	
Patient pregnant (62…00)	2	Patient pregnant (62…00)	6
Patient ? pregnant (6219.00)	1	A/N care: obstetric risk (623..00)	3
Born by normal vaginal delivery (14Y3.00)	1	Birth details (63…00)	3
Perinatal conditions (Q….00)	1	-	-
**Conditions originating in the perinatal period**		**Conditions originating in the perinatal period**	
Fetus and newborn affected by maternal use of alcohol (PK83.00)	1	Periventricular leucomalacia (Q48E.00)	2
**Congenital malformations**		**Congenital malformations**	
Congenital curly toes (PF66500)	2	Congenital curly toes (PF66500)	10
Arachnoid cyst (F280000)	2	Congenital genu valgum – knock-knee (PF64100)	7
Congenital hammer toe (PF66200)	2	Pectus excavatum, congenital (PE80.00)	4
Pectus excavatum, congenital (PE80.00)	2	-	-
**Symptoms, signs and abnormal clinical/lab findings**		**Symptoms, signs and abnormal clinical/lab findings**	
Feels hot/feverish (1652.00)	96	Feels hot/feverish (1652.00)	439
Chest pain (182..00)	94	Chest pain (182..00)	327
Feels unwell (16E..00)	81	Feels unwell (16E..00)	313
**Injury and other external causes**		**Injury and other external causes**	
Seen in minor injuries department (9N0i.00)	116	Seen in minor injuries department (9N0i.00)	402
Dressing of wound (81H..00)	113	Dressing of wound (81H..00)	346
Fall – accidental (TC…11)	86	Fall – accidental (TC…11)	287
**Factors influencing health status/contact with services**		**Factors influencing health status/contact with services**	
Telephone encounter (9N31.00)	469	Notes summary on computer (9344.00)	2379
Letter from specialist (9N36.00)	452	Telephone encounter (9N31.00)	2187
Seen in paediatric clinic (9N1V.00)	359	Seen in hospital casualty (9N19.00)	1740
**Dyslexia, dyspraxia or educational psychology**		**Dyslexia, dyspraxia or educational psychology**	
Dyslexia (ZS32.00)	30	Dyspraxia (27E5111)	23
[D] Dyslexia (R046300)	25	Dyslexia (ZS32.00)	14
Dyspraxia (27E5111)	19	[D] Dyslexia (R046300)	11

Supplementary Table 5 Most commonly occurring first GP attendance Drug codes within each category of prescriptions for children and young people with and without ADHD (n=48,263)

	Cases (*n*=8,127)		Controls (*n*=40,136)
**GP prescriptions (Drug codes), *n***			
**Gastro-intestinal system**		**Gastro-intestinal system**	
Prednisolone 5mg soluble tablets (955)	176	Prednisolone 5mg soluble tablets (955)	687
Lactulose 3.1-3.7g/5ml oral solution (4613)	142	Lactulose 3.1-3.7g/5ml oral solution (4613)	461
Movicol Paediatric Plain oral powder 6.9g sachets (Norgine Pharmaceuticals Ltd) (6599)	115	Movicol Paediatric Plain oral powder 6.9g sachets (Norgine Pharmaceuticals Ltd) (6599)	350
**Cardiovascular**		**Cardiovascular**	
Tranexamic acid 500mg tablets (1074)	15	Tranexamic acid 500mg tablets (1074)	36
Aspirin 75mg dispersible tablets (3)	5	Aspirin 75mg dispersible tablets (3)	9
MaxEPA 1g capsules (Seven Seas Ltd) (2662)	3	Atenolol 25mg tablets (26)	6
**Respiratory system**		**Respiratory system**	
Salbutamol 100micrograms/dose inhaler CFC free (17)	590	Salbutamol 100micrograms/dose inhaler CFC free (17)	2293
Cetirizine 1mg/ml oral solution sugar free (2916)	205	Cetirizine 1mg/ml oral solution sugar free (2916)	972
Chlorphenamine 2mg/5ml oral solution (1549)	150	Chlorphenamine 2mg/5ml oral solution (1549)	690
**Central nervous system**		**Central nervous system**	
Paracetamol 250mg/5ml oral suspension sugar free (4186)	277	Paracetamol 250mg/5ml oral suspension sugar free (4186)	1099
Paracetamol 250mg/5ml oral suspension (262)	213	Paracetamol 250mg/5ml oral suspension (262)	836
Paracetamol 250mg/5ml oral suspension sugar free (1689)	135	Paracetamol 250mg/5ml oral suspension sugar free (1689)	581
**Infections**		**Infections**	
Amoxicillin 125mg/5ml oral suspension sugar free (503)	516	Amoxicillin 125mg/5ml oral suspension sugar free (503)	2579
Amoxicillin 250mg/5ml oral suspension sugar free (585)	411	Amoxicillin 250mg/5ml oral suspension sugar free (585)	1755
Amoxicillin 125mg/5ml oral suspension (62)	356	Amoxicillin 125mg/5ml oral suspension (62)	1307
**Endocrine system**		**Endocrine system**	
Desmopressin 200microgram tablets (4301)	55	Desmopressin 200microgram tablets (4301)	114
Desmopressin 120microgram oral lyophilisates sugar free (13218)	20	Desmopressin 120microgram oral lyophilisates sugar free (13218)	45
Desmopressin 10micrograms/dose nasal spray (5878)	16	Desmopressin 10micrograms/dose nasal spray (5878)	32
**Obstetrics, gynaecological and urinary tract disorders**		**Obstetrics, gynaecological and urinary tract disorders**	
Clotrimazole 1% cream (1007)	167	Clotrimazole 1% cream (1007)	685
DesmoMelt 120microgram oral lyophilisates (Ferring Pharmaceuticals Ltd) (7240)	36	Microgynon 30 tablets (Bayer Plc) (41)	74
Microgynon 30 tablets (Bayer Plc) (41)	28	Norethisterone 5mg tablets (1083)	68
**Malignant disease and immunosuppression**		**Malignant disease and immunosuppression**	
Neoral 100mg/ml oral solution (Novartis Pharmaceuticals UK Ltd) (1905)	1	Tacrolimus 1mg capsules (2839)	3

	Cases (*n*=8,127)		Controls (*n*=40,136)
Zoladex 3.6mg implant SafeSystem pre-filled syringes (AstraZeneca UK Ltd) (2187)	1	Tacrolimus 500microgram capsules (6495)	1
Tacrolimus 1mg capsules (2839)	1	Ciclosporin 50mg capsules (2837)	1
-	-	Neoral 100mg/ml oral solution (Novartis Pharmaceuticals UK Ltd) (1905)	1
**Nutrition and blood**		**Nutrition and blood**	
Dioralyte Sachets (Aventis Pharma) (866)	67	Dioralyte Sachets (Aventis Pharma) (866)	271
Sytron oral solution (Forum Health Products Ltd) (1534)	30	Dioralyte oral powder sachets blackcurrant (Sanofi) (41361)	141
Dioralyte oral powder sachets blackcurrant (Sanofi) (41361)	21	Sytron oral solution (Forum Health Products Ltd) (1534)	60
**Musculoskeletal and connective tissue**		**Musculoskeletal and connective tissue**	
Ibuprofen 100mg/5ml Oral suspension (2938)	312	Ibuprofen 100mg/5ml Oral suspension (2938)	1223
Ibuprofen 100mg/5ml oral suspension (647)	190	Ibuprofen 100mg/5ml oral suspension (647)	775
Ibuprofen 200mg tablets (416)	75	Ibuprofen 200mg tablets (416)	344
**Eye**		**Eye**	
Chloramphenicol 0.5% eye drops (79)	316	Chloramphenicol 0.5% eye drops (79)	1422
Sodium cromoglicate 2% eye drops (1459)	191	Sodium cromoglicate 2% eye drops (1459)	1018
Chloramphenicol 1% eye ointment (170)	129	Chloramphenicol 1% eye ointment (170)	555
**Ear, nose and oropharynx**		**Ear, nose and oropharynx**	
Naseptin nasal cream (Alliance Pharmaceuticals Ltd) (975)	119	Beclometasone 50micrograms/dose nasal spray (88)	518
Beclometasone 50micrograms/dose nasal spray (88)	103	Naseptin nasal cream (Alliance Pharmaceuticals Ltd) (975)	431
Beconase Aqueous 50micrograms/dose nasal spray (GlaxoSmithKline UK Ltd) (888)	83	Beconase Aqueous 50micrograms/dose nasal spray (GlaxoSmithKline UK Ltd) (888)	409
**Skin**		**Skin**	
Hydrocortisone 1% cream (73)	205	Fusidic acid 2% cream (1003)	779
Fusidic acid 2% cream (1003)	203	Hydrocortisone 1% cream (73)	776
Fusidin 2% cream (LEO Pharma) (336)	195	Aqueous cream (59)	722
**Immunological products and vaccines**		**Immunological products and vaccines**	
Typhim Vi 25micrograms/0.5ml vaccine solution for injection pre-filled syringes (sanofi pasteur MSD Ltd) (869)	38	Typhim Vi 25micrograms/0.5ml vaccine solution for injection pre-filled syringes (sanofi pasteur MSD Ltd) (869)	341
Influenza vaccine (split virion, inactivated) suspension for injection 0.5ml pre-filled syringes (639)	36	Havrix monodose 720 unit/0.5ml Kabiquick (GlaxoSmithKline UK Ltd) (2353)	209
Havrix monodose 720 unit/0.5ml Kabiquick (GlaxoSmithKline UK Ltd) (2353)	31	Influenza vaccine (split virion, inactivated) suspension for injection 0.5ml pre-filled syringes (639)	156
**Anaesthesia**		**Anaesthesia**	
Emla 5% cream (AstraZeneca UK Ltd) (263)	226	Emla 5% cream (AstraZeneca UK Ltd) (263)	546
Ametop 4% Gel (Smith & Nephew Healthcare Ltd) (5883)	20	Ametop 4% Gel (Smith & Nephew Healthcare Ltd) (5883)	45
Lidocaine 2.5% / Prilocaine 2.5% cream (4786)	12	Lidocaine 2.5% / Prilocaine 2.5% cream (4786)	30

	Cases (*n*=8,127)		Controls (*n*=40,136)
**Other drugs and preparations**		**Other drugs and preparations**	
OneTouch Ultra testing strips (LifeScan) (5763)	5	OneTouch Ultra testing strips (LifeScan) (5763)	16
Betadine 4% shampoo (Molnlycke Health Care Ltd) (7743)	3	Sharpsguard disposal unit Yellow (Daniels Healthcare Ltd) (38572)	10
Chlorhexidine gluconate 0.12% mouthwash sugar free (5623)	2	Sharpsguard disposal unit Orange (Daniels Healthcare Ltd) (38590)	6
**Dressings**		**Dressings**	
Tegaderm Film dressing 6cm x 7cm (3M Health Care Ltd) (4894)	16	Tegaderm Film dressing 6cm x 7cm (3M Health Care Ltd) (4894)	50
Micropore tape 2.5cm (3M Health Care Ltd) (1055)	9	Mepore dressing 7cm x 8cm (Molnlycke Health Care Ltd) (5705)	42
Jelonet dressing 10cm x 10cm (Smith & Nephew Healthcare Ltd) (435)	9	Steri-strip skin closure strips 6mm x 75mm (3M Health Care Ltd) (3177)	28
**Appliances**		**Appliances**	
Softclix lancets 0.4mm/28gauge (Roche Diabetes Care Ltd) (4929)	3	FreeStyle Optium testing strips (Abbott Laboratories Ltd) (6053)	10
FreeStyle testing strips (Abbott Laboratories Ltd) (5961)	2	Softclix lancets 0.4mm/28gauge (Roche Diabetes Care Ltd) (4929)	10
Aquagel lubricating jelly (Ecolab Healthcare Division) (14351)	2	FreeStyle testing strips (Abbott Laboratories Ltd) (5961)	9
**Incontinence appliances**		**Incontinence appliances**	
-	-	Simpla S2 non-drainable night drainage bag 320902 2litre (Coloplast Ltd) (1507)	2
-	-	Bladder irrigating syringe 100ml (4173)	2
**Stoma appliances**		**Stoma appliances**	
Assura Inspire Soft Seal convex ileostomy bag with Hide-Away outlet, midi 14404 Starter hole 15mm-33mm Opaque (Coloplast Ltd) (16456)	1	Simpla S2 non-drainable night drainage bag 320902 2litre (Coloplast Ltd) (41838)	2
Stopper for washout set AP10/60 10Fr, 60mm (Medicina Ltd) (28670)	1	Bladder irrigating syringe 100ml (1507)	2
Stopper for washout set AP10/30 10Fr, 30mm (Medicina Ltd) (23930)	1	URIbag pocket sized male urinal URIbag 1.1litre (Manfred Sauer UK Ltd) (4173)	2
**Homeopathy**		**Homeopathy**	
Hepar sulfuris 30c homeopathic tablets (11014)	1	Arnica montana 30c homeopathic tablets (11051)	2
Belladonna 30c homeopathic tablets (11028)	1	Calcarea carbonica 30c homeopathic tablets (11022)	2
Ignatia amara 30c homeopathic tablets (11077)	1	Plantago compound oint (23169)	1
Medorrhinum (11078)	1	Argentum nitricum 30c homeopathic tablets (11015)	1
Pulsatilla nigricans 30c tablet (11081	1	Phosphorus 30c homeopathic tablets (11470)	1
Ipecacuanha 6c homeopathic tablets (15112)	1	Arsenicum album 30c homeopathic pillules (30682)	1
Antimonium cruduc 30c tablets (21483)	1	Kalium muriaticum 30c Tablet (30358)	1
Nux vomica 6c homeopathic tablets (23297)	1	Nux vomica 6c homeopathic tablets (23297)	1
Calendula officinalis 0.45% / Hypericum perforatum 0.45% cream (31075)	1	Arsenicum album 30c homeopathic tablets (11130)	1
Skin tone lotion (Weleda (UK) Ltd) (40495)	1	Kalium muriaticum 6x homeopathic tablets (53146)	1

	Cases (*n*=8,127)		Controls (*n*=40,136)
Urtica dioica ferro culta with prunus spinosa D3 + D4 oral drops (42773)	1	Natrum muriaticum 30c homeopathic tablets (11302)	1
-	-	Arsenicum album 6c homeopathic tablets (19295)	1
		Belladonna 30c homeopathic tablets (11028)	
-	-	Thuja occidentalis 6c homeopathic tablets (15704)	1
-	-	Menodoron drops (Weleda (UK) Ltd) (41260)	1
**Unknown**		**Unknown**	
* (1)	1	* (1)	282

Supplementary Table 6 Most commonly occurring first attendance hospital diagnosis codes within each category of diagnosis for children and young people with and without ADHD (n=48,263)

	Cases (*n*=8,127)		Controls (*n*=40,136)
**Hospital diagnoses (ICD10 codes), *n***			
**Infectious and parasitic disease**		**Infectious and parasitic diseases**	
Viral infection, unspecified (B34.9)	42	Viral infection, unspecified (B34.9)	151
Viral intestinal infection, unspecified (A08.4)	12	Viral intestinal infection, unspecified (A08.4)	45
Varicella without complication (B01.9)	6	Other viral agents as the cause of diseases classified to other chapters (B97.8)	25
**Neoplasms, cancers and diseases of the blood**		**Neoplasms, cancers and diseases of the blood**	
Allergic purpura (D69.0)	6	Allergic purpura (D69.0)	24
Iron deficiency anaemia, unspecified (D50.9)	5	Acute lymphoblastic leukaemia [ALL] (C91.0)	9
Anaemia, unspecified (D64.9)	5	Anaemia, unspecified (D64.9)	7
**Endocrine**		**Endocrine**	
Obesity, unspecified (E66.9)	8	Volume depletion (E86)	32
Insulin-dependent diabetes mellitus (E10.9)	6	Insulin-dependent diabetes mellitus (E10.9)	26
Short stature, not elsewhere classified (E34.3)	4	Cystic fibrosis, unspecified (E84.9)	11
**Mental and behavioural disorders**		**Mental and behavioural disorders**	
Disturbance of activity and attention (F90.0)	74	Developmental disorder of scholastic skills, unspecified (F81.9)	31
Childhood autism (F84.0)	52	Childhood autism (F84.0)	29
Developmental disorder of scholastic skills, unspecified (F81.9)	31	Developmental disorder of speech and language, unspecified (F80.9)	11
**Diseases of the nervous system**		**Diseases of the nervous system**	
Epilepsy, unspecified (G40.9)	27	Sleep apnoea (G47.3)	47
Generalized idiopathic epilepsy and epileptic syndrome (G40.3)	17	Epilepsy, unspecified (G40.9)	34
Sleep apnoea (G47.3)	15	Cerebral palsy, unspecified (G80.9)	12
**Eye, ear, nose and throat, mouth and dental**		**Eye, ear, nose and throat, mouth and dental**	
Chronic mucoid otitis media (H65.3)	81	Chronic mucoid otitis media (H65.3)	180
Otitis media, unspecified (H66.9)	21	Otitis media, unspecified (H66.9)	50
Hearing loss, unspecified (H91.9)	16	Nonsuppurative otitis media, unspecified (H65.9)	44
**Circulatory system**		**Circulatory system**	
Nonspecific mesenteric lymphadenitis (I88.0)	12	Nonspecific mesenteric lymphadenitis (I88.0)	24
Nonspecific lymphadenitis, unspecified (I88.9)	3	Supraventricular tachycardia (I47.1)	8
Cardiomegaly (I51.7)	2	Nonspecific lymphadenitis, unspecified (I88.9)	5
Scrotal varices (I86.1)	2	-	-
Essential (primary) hypertension (I10)	2	-	-
**Respiratory**		**Respiratory**	
Asthma, unspecified (J45.9)	146	Asthma, unspecified (J45.9)	356
Acute tonsillitis, unspecified (J03.9)	44	Acute tonsillitis, unspecified (J03.9)	173
Chronic tonsillitis (J35.0)	30	Acute upper respiratory infection, unspecified (J06.9)	75
**Gastroenterology**		**Gastroenterology**	
Dental caries, unspecified (K02.9)	116	Dental caries, unspecified (K02.9)	285

	Cases (*n*=8,127)		Controls (*n*=40,136)
Constipation (K59.0)	27	Constipation (K59.0)	71
Noninfective gastroenteritis and colitis, unspecified (K52.9)	23	Noninfective gastroenteritis and colitis, unspecified (K52.9)	60
**Skin**		**Skin**	
Dermatitis, unspecified (L30.9)	18	Dermatitis, unspecified (L30.9)	49
Cellulitis of other parts of limb (L03.1)	6	Ingrowing nail (L60.0)	13
Local infection of skin and subcutaneous tissue, unspecified (L08.9)	5	Cellulitis of other parts of limb (L03.1)	9
Urticaria, unspecified (L50.9)	5	-	-
**Musculoskeletal and connective tissue**		**Musculoskeletal and connective tissue**	
Pain in joint (M25.5)	10	Paint in joint (M25.5)	25
Pain in limb (M79.6)	5	Other specified joint derangements, not elsewhere classified (M24.8)	15
Nonunion of fracture [pseudarthrosis] (M84.1)	3	Pain in limb (M79.6)	12
**Genitourinary including gynaecology**		**Genitourinary including gynaecology**	
Redundant prepuce, phimosis and paraphismosis (N47)	45	Redundant prepuce, phimosis and paraphismosis (N47)	187
Urinary tract infection, site not specified (N39.0)	11	Other specified disorders of male genital organs (N50.8)	29
Torsion of testis (N44)	6	Hydrocele, unspecified (N43.3)	26
**Pregnancy, childbirth, puerperium**		**Pregnancy, childbirth, puerperium**	
Medical abortion (O04.9)	2	Medical abortion (O04.9)	3
**-**	-	Eclampsia in labour (O15.1)	1
-	-	Maternal care for poor fetal growth (O36.5)	1
-	-	Prolonged second stage (of labour) (O63.1)	1
-	-	Spontaneous abortion (O03.4)	1
-	-	Haemorrhage in early pregnancy, unspecified (O20.9)	1
-	-	Spontaneous abortion (O03.9)	1
**-**	-	Maternal care for fetal problem, unspecified (O36.9)	1
**Conditions originating in perinatal period**		**Conditions originating in perinatal period**	
Congenital hydrocele (P83.5)	2	Congenital hydrocele (P83.5)	6
Other preterm infants (P07.3)	1	Congenital hyptonia (P94.2)	2
Erb’s paralysis due to birth injury (P14.0)	1	Unspecified chronic respiratory disease originating in the perinatal period (P27.9)	2
Congenital renal failure (P96.0)	1	Congenital cytomegalovirus infection (P35.1)	2
Unspecified chronic respiratory disease originating in the perinatal period (P27.9)	1	-	-
**Congenital malformations**		**Congenital malformations**	
Prominent ear (Q17.5)	10	Prominent ear (Q17.5)	33
Undescended testicle, unilateral (Q53.1)	8	Undescended testicle, unilateral (Q53.1)	32
Other congenital malformations of testis and scrotum (Q55.2)	7	Ankyloglossia (Q38.1)	21
**Symptoms, signs and abnormal clinical/lab findings**		**Symptoms, signs and abnormal clinical/lab findings**	
Other and unspecified abdominal pain (R10.4)	30	Other and unspecified abdominal pain (R10.4)	120

	Cases (*n*=8,127)		Controls (*n*=40,136)
Other and unspecified convulsions (R56.8)	30	Nausea and vomiting (R11)	76
Nausea and vomiting (R11)	27	Wheezing (R06.2)	72
**Injury and other external causes**		**Injury and other external causes**	
Unspecified injury of head (S09.9)	24	Fracture of lower end of both ulna and radius (S52.6)	76
Fracture of lower end of radius (S52.5)	17	Fracture of lower end of radius (S52.5)	73
Poisoning: 4-Aminophenol derivatives (T39.1)	17	Unspecified injury of head (S09.9)	64
**External causes of morbidity and mortality**		**External causes of morbidity and mortality**	
Exposure to unspecified factor (X59.9)	22	Exposure to unspecified factor (X59.9)	59
Pedestrian injured in collision with car, pick-up truck or van (V03.1)	11	Fall involving playground equipment (W09.9)	33
Foreign body entering into or through eye or natural orifice (W44.9)	11	Foreign body entering into or through eye or natural orifice (W44.9)	27
-	-	Unspecified fall (W19.9)	27
**Codes for special purposes**		**Codes for special purposes**	
Methicillin resistant agent (U80.1)	1	Methicillin resistant agent (U80.1)	1
**Factors influencing health status/contact with services**		**Factors influencing health status/contact with services**	
Follow-up care involving removal of fracture plate and other internal fixation device (Z47.0)	21	Follow-up care involving removal of fracture plate and other internal fixation device (Z47.0)	57
Personal history of allergy to penicillin (Z88.0)	18	Personal history of allergy to penicillin (Z88.0)	54
Personal history of self-harm (Z91.5)	18	Procedure not carried out for other reasons (Z53.8)	48

Supplementary Table 7 Most commonly occurring first attendance hospital procedure codes under broad OPCS4 categories for children and young people with and without ADHD (n=48,263)

	Cases (*n*=8,127)		Controls (*n*=40,136)
**Hospital procedures (OPCS-4 codes), *n***			
**Nervous system**		**Nervous system**	
Neurophysiological operations (A847)	14	Diagnostic spinal puncture (A559)	24
Neurophysiological operations (A841)	12	Neurophysiological operations (A847)	11
Diagnostic spinal puncture (A559)	7	Neurophysiological operations (A841)	6
**Endocrine system and breast**		**Endocrine system and breast**	
Operations on thyroglossal tissue (B101)	1	Operations on thyroglossal tissue (B101)	2
Operations on thyroglossal tissue (B108)	1	Excision of thyroid gland (B081)	1
-	-	Operation on nipple and areola (B353)	1
-	-	Total excision of breast (B275)	1
-	-	Total excision of breast (B272)	1
**Respiratory tract**		**Respiratory tract**	
Operations on adenoid (E201)	79	Operations on adenoid (E201)	172
Other operations on pharynx (E276)	17	Other operations on pharynx (E276)	35
Surgical arrest of bleeding from internal nose (E051)	11	Operations on adenoid (E204)	30
**Skin**		**Skin**	
Suture of skin of head or neck (S411)	11	Other excision of lesion of skin (S065)	27
Suture of skin of other site (S421)	10	Exploration of other skin of other site (S571)	23
Other excision of lesion of skin (S065)	9	Other excision of lesion of skin (S069)	23
-	-	Suture of skin of other site (S069)	23
**Soft tissue**		**Soft tissue**	
Simple excision of inguinal hernial sac (T192)	11	Simple excision of inguinal hernial sac (T192)	33
Adjustment to length of tendon (T705)	7	Simple excision of inguinal hernial sac (T193)	16
Primary repair of tendon (T676)	5	Adjustment to length of tendon (T705)	13
**Diagnostic image testing**		**Diagnostic image testing**	
Diagnostic imaging of central nervous system (U052)	34	Diagnostic imaging of central nervous system (U051)	53
Diagnostic imaging of central nervous system (U051)	17	Diagnostic imaging of central nervous system (U052)	41
Other diagnostic tests (U331)	11	Other diagnostic tests (U331)	15
**Bones and joints**		**Bones and joints**	
Other closed reduction of fracture of bone (W262)	49	Other closed reduction of fracture of bone (W262)	186
Reduction of fracture of other bone of face (V092)	9	Closed reduction of fracture of bone and internal fixat (W243)	26
Closed reduction of fracture of bone and internal fixat (W243)	6	Closed reduction of fracture of bone and internal fixat (W242)	24
**Miscellaneous**		**Miscellaneous**	
Anaesthetic without surgery (X598)	9	Immobilisation using plaster cast (X481)	30
High cost neurology drugs (X851)	6	Continuous infusion of therapeutic substance (X292)	20
Blood withdrawal (X369)	6	High cost neurology drugs (X851)	14
-	-	Other intravenous injection (X352)	14
**Ear, nose, mouth and dental**		**Ear, nose, mouth and dental**	
Simple extraction of tooth (F104)	114	Simple extraction of tooth (F104)	307
Drainage of middle ear (D151)	107	Drainage of middle ear (D151)	292

	Cases (*n*=8,127)		Controls (*n*=40,136)
Excision of tonsil (F341)	65	Excision of tonsil (F341)	198
**Gastroenterology**		**Gastroenterology**	
Emergency excision of appendix (H012)	17	Emergency excision of appendix (H012)	83
Diagnostic fibreoptic endoscopic exam/upper gastrointe (G451)	8	Diagnostic fibreoptic endoscopic exam/upper gastrointe (G451)	25
Other excision of appendix (H029)	5	Other excision of appendix (H029)	10
**Circulatory**		**Circulatory**	
Other vein related operations (L912)	3	Other vein related operations (L913)	11
Transluminal operations on abnormality of great vessel (L031)	2	Other vein related operations (L912)	8
Contrast radiology of the heart (K631)	2	Other vein related operations (L911)	6
Other vein related operations (L916)	2	-	-
Other vein related operations (L915)	2	-	-
**Genitourinary**		**Genitourinary**	
Operations on prepuce (N303)	42	Operations on prepuce (N303)	172
Other placement of testis in scrotum (N092)	8	Operations on prepuce (N302)	26
Extirpation of lesion of testis (N071)	5	Other placement of testis in scrotum (N092)	25
Bilateral placement of testes in scrotum (N082)	5	-	-
**Subsidiary classification of methods**		**Subsidiary classification of methods**	
Radiology procedures (Y981)	45	Radiology procedures (Y981)	112
General anaesthetic (Y809)	26	General anaesthetic (Y809)	60
Approach to organ under image control (Y535)	15	Approach to organ under image control (Y535)	55
**Subsidiary classification of sites**		**Subsidiary classification of sites**	
Laterality of operation (Z941)	231	Laterality of operation (Z941)	572
Laterality of operation (Z942)	116	Laterality of operation (Z942)	403
Laterality of operation (Z943)	110	Laterality of operation (Z943)	373
