# Supplementary material for: Use of healthcare services before diagnosis of attention-deficit/hyperactivity disorder: a population-based matched case-control study
Source: Arch Dis Child. 2023 Oct 30;109(1):46–51. doi: 10.1136/archdischild-2023-325637 (PMC10803994; doi:10.1136/archdischild-2023-325637)

Attention-deficit/hyperactivity disorder and healthcare service utilisation: a case control study: Supplementary tables

Supplementary figure 1 Admissions to hospital by categories of procedures (OPCS4 codes), comparing children and young people with (n=8,127) vs. without (n=40,136) ADHD (n=48,263)

Reasons for operation

	ADHD		Controls				
**Diagnosis category**	**Freq.**	**%**	**Freq.**	**%**	**OR**	**LL**	**UL**
Subsidiary sites	612	7.5	1,835	4.6	1.71	1.55	1.88
Eye, ENT, oral	496	6.1	1,399	3.5	1.81	1.63	2.01
Subsidiary methods	218	2.7	538	1.3	2.04	1.74	2.39
Gastroenterology	210	0.5	63	0.8	1.50	1.13	1.99
Respiratory	155	1.9	375	0.9	2.08	1.72	2.52
Bones and joints	131	1.6	496	1.2	1.31	1.08	1.59
Image testing, rehabilitation	106	1.3	230	0.6	2.31	1.83	2.92
Genitourinary	98	1.2	397	1.0	1.22	0.98	1.53
Skin	95	1.2	259	0.7	1.82	1.44	2.30
Miscellaneous operations	59	0.7	194	0.5	1.51	1.13	2.03
Nervous system	54	0.7	76	0.2	3.48	2.45	4.95
Soft tissue	53	0.7	154	0.4	1.70	1.24	2.32
Circulatory	20	0.3	54	0.1	1.83	1.10	3.06

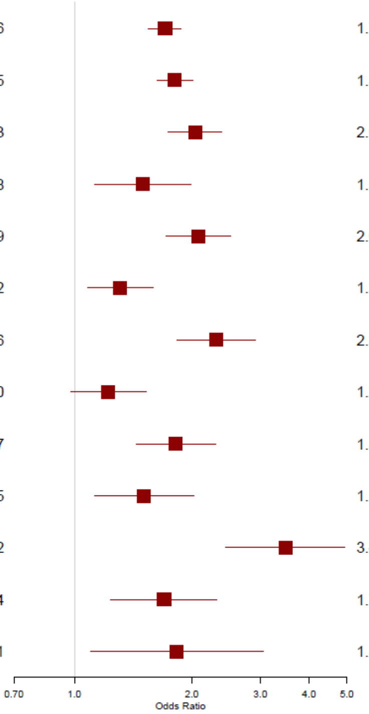

Notes: Categories are arranged in order of frequency for CYP with ADHD. Freq. – Frequency; OR – Odds ratio; LL – Lower limit of the 95%CI; UL – Upper limit of the 95%CI.

Supplementary figure 2 Attendances to the GP by categories of diagnoses (Read codes), comparing children and young people with vs. without ADHD by sex (n=48,263)

Reasons for attendance

■ Male ● Female

Diagnosis category	Males OR	Males LL	Males UL	Females OR	Females LL	Females UL
Non−specific	5.16	4.57	5.82	6.98	5.15	9.67
Eye, ENT, oral	1.38	1.31	1.45	1.64	1.46	1.84
Skin	1.25	1.18	1.32	1.47	1.31	1.65
Respiratory	1.30	1.24	1.38	1.50	1.34	1.69
Gastroenterology	1.30	1.22	1.39	1.38	1.20	1.59
Musculoskeletal	1.29	1.20	1.38	1.72	1.49	1.98
Injury and external	1.66	1.55	1.77	2.11	1.82	2.44
Mental / behavioural	21.26	19.88	22.74	26.26	22.51	30.64
Genitourinary	1.49	1.39	1.62	1.66	1.45	1.90
Symptoms and signs	1.23	1.10	1.34	1.42	1.15	1.75
Nervous system	1.97	1.80	2.15	2.16	1.78	2.60
Infections	1.23	1.11	1.37	1.29	1.04	1.60
Circulatory	1.39	1.16	1.68	1.32	0.87	1.96
Dyslexia, dyspraxia etc	6.90	5.30	9.01	15.66	7.16	37.79
Endocrine	1.32	0.92	1.86	2.56	1.34	4.76
Neoplams	2.14	1.33	3.36	2.29	0.71	6.46
Congenital malformations	1.94	1.13	3.24	4.25	1.18	14.79

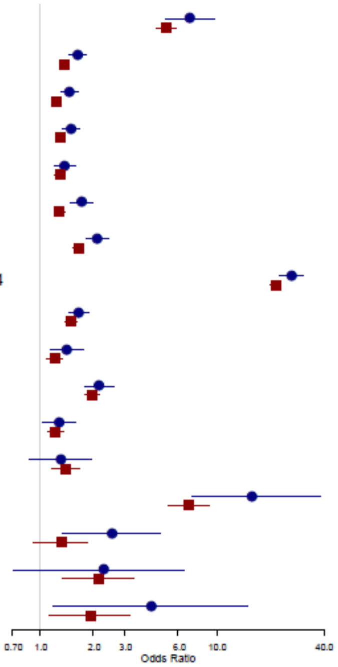

Supplementary figure 3 Attendances to the GP by categories of prescriptions (drug codes), comparing children and young people with vs. without ADHD by sex (n=48,263)

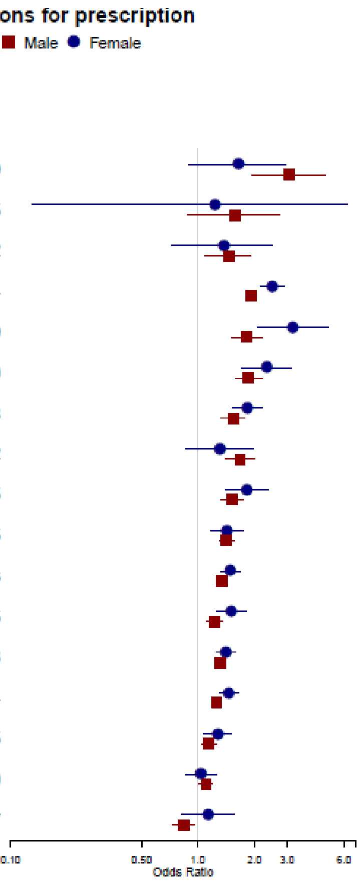

	Males			Females		
**Diagnosis category**	**OR**	**LL**	**UL**	**OR**	**LL**	**UL**
Circulatory	3.06	1.93	4.79	1.65	0.89	2.92
Non−specific	1.59	0.88	2.75	1.24	0.13	6.20
Unknown	1.46	1.09	1.92	1.38	0.72	2.48
Nervous system	1.91	1.79	2.04	2.50	2.18	2.86
Endocrine	1.81	1.50	2.19	3.22	2.09	4.91
Anaesthesia	1.87	1.59	2.20	2.33	1.72	3.13
Obstetrics, gynaecological etc.	1.54	1.33	1.78	1.84	1.53	2.22
Dressings	1.68	1.40	2.02	1.31	0.86	1.96
Nutrition and blood	1.53	1.33	1.75	1.83	1.41	2.37
Gastroenterology	1.42	1.30	1.55	1.43	1.18	1.73
Respiratory	1.35	1.27	1.43	1.49	1.32	1.69
Musculoskeletal	1.23	1.12	1.35	1.51	1.27	1.81
Infections	1.31	1.24	1.38	1.41	1.25	1.58
Skin	1.26	1.20	1.34	1.46	1.30	1.64
Ear, nose and oropharynx	1.15	1.06	1.25	1.28	1.08	1.52
Eye	1.11	1.02	1.20	1.04	0.86	1.25
Vaccines etc.	0.84	0.73	0.97	1.14	0.82	1.56

Supplementary figure 4 Medical conditions recorded in the GP medical records, comparing children and young people with vs. without ADHD by sex (n=48,263)

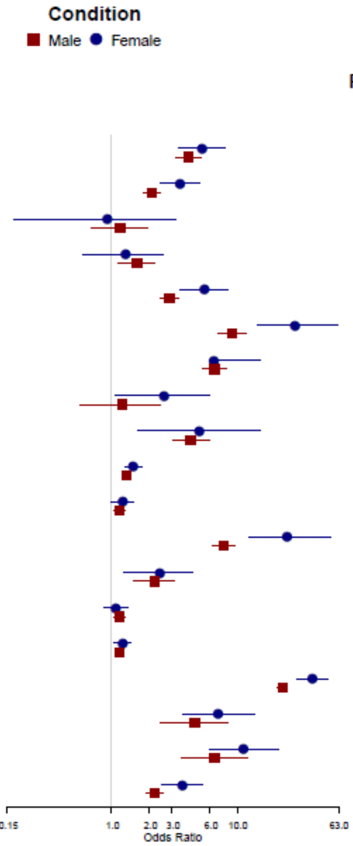

	Males			Females		
Diagnosis category	OR	LL	UL	OR	LL	UL
Epilepsy	4.11	3.26	5.17	5.19	3.38	7.96
Incontinence	2.11	1.81	2.46	3.49	2.45	4.95
Diabetes	1.18	0.70	1.92	0.93	0.17	3.24
Migraines	1.60	1.14	2.21	1.30	0.60	2.57
Language impairment	2.88	2.45	3.38	5.42	3.53	8.31
Learning disability	8.95	6.91	11.63	28.24	14.20	62.25
Sleep disorders	6.53	5.29	8.08	6.43	5.95	15.16
Alcohol abuse	1.23	0.57	2.43	2.61	1.08	5.92
Tic disorders	4.25	3.04	5.92	4.96	1.62	15.19
Asthma	1.31	1.23	1.40	1.49	1.28	1.74
Abdominal pain	1.16	1.04	1.29	1.23	1.00	1.50
Autism	7.72	6.32	9.45	24.52	12.20	54.45
Anxiety	2.20	1.51	3.18	2.42	1.27	4.45
Eczema	1.16	1.04	1.29	1.08	0.87	1.35
Dermatitis	1.16	1.08	1.24	1.23	1.05	1.42
Behaviour codes	22.49	20.52	24.66	38.84	29.55	51.60
Depression	4.56	2.47	8.37	6.97	3.64	13.59
Self−harm	6.53	3.62	11.94	11.02	6.02	20.91
Enuresis	2.21	1.90	2.58	3.63	2.51	5.23

Supplementary figure 5 Admissions to hospital by categories of diagnoses (ICD10 codes), comparing children and young people with vs. without ADHD by sex (n=48,263)

Reasons for attendance

Male ● Female

	Males			Females		
**Diagnosis category**	**OR**	**LL**	**UL**	**OR**	**LL**	**UL**
Non−specific	1.73	1.47	2.03	4.08	3.07	5.40
Eye, ENT, oral	1.89	1.59	2.25	2.91	2.00	4.21
Skin	1.61	1.16	2.21	3.02	1.55	5.74
Respiratory	1.65	1.44	1.89	2.90	2.15	3.88
Gastroenterology	1.69	1.46	1.96	1.87	1.35	2.56
Musculoskeletal	1.37	0.99	1.83	1.70	0.80	3.36
Injury and external	1.71	1.49	1.97	4.28	3.17	5.75
Mental / behavioural	8.93	7.14	11.22	16.72	10.30	28.07
Genitourinary	1.15	0.90	1.46	3.48	1.62	7.26
Symptoms and signs	1.91	1.66	2.19	2.97	2.26	3.88
Nervous system	2.86	2.19	3.73	6.53	3.97	10.84
Infections	1.26	0.97	1.63	2.65	1.58	4.37
Circulatory	2.28	1.41	3.60	3.16	1.03	8.93
Endocrine	1.02	0.64	1.57	2.12	0.85	4.85
Neoplams	1.29	0.86	1.89	0.96	0.33	2.33
Congenital malformations	1.73	1.37	2.17	2.36	1.26	4.25
External causes	1.67	1.45	1.93	4.07	3.01	5.50

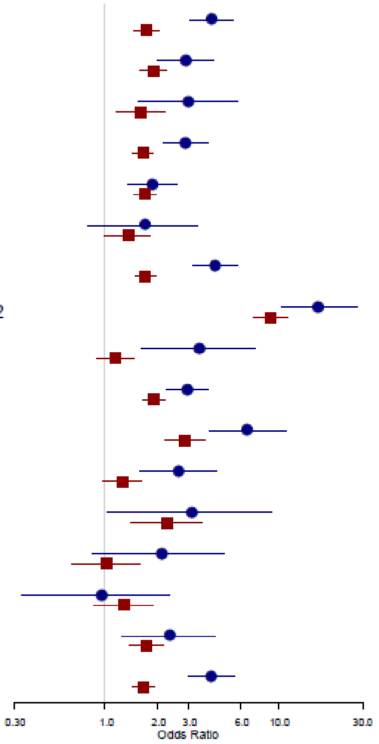

Supplementary figure 6 Admissions to hospital by categories of procedures (OPCS4 codes), comparing children and young people with vs. without ADHD by sex (n=48,263)

Reasons for operation

	Males			Females		
**Diagnosis category**	**OR**	**LL**	**UL**	**OR**	**LL**	**UL**
Eye, ENT, oral	1.71	1.52	1.92	2.26	1.76	2.89
Skin	1.71	1.30	2.23	2.49	1.35	4.45
Respiratory	1.89	1.51	2.34	2.97	1.90	4.57
Gastroenterology	1.29	0.91	1.79	2.56	1.34	4.76
Genitourinary	1.17	0.92	1.48	2.67	0.90	7.20
Nervous system	3.12	2.03	4.73	5.34	2.40	11.96
Circulatory	1.84	0.97	3.33	1.80	0.42	6.09
Soft tissue	1.69	1.17	2.39	1.80	0.69	4.21
Image testing, rehab.	2.18	1.66	2.84	2.80	1.60	4.80
Bones and joints	1.20	0.96	1.49	2.22	1.31	3.66
Miscellaneous operations	1.55	1.12	2.13	1.19	0.40	2.97
Subsidiary methods	1.98	1.66	2.37	2.28	1.51	3.38
Subsidiary sites	1.57	1.41	1.74	2.53	2.01	3.19

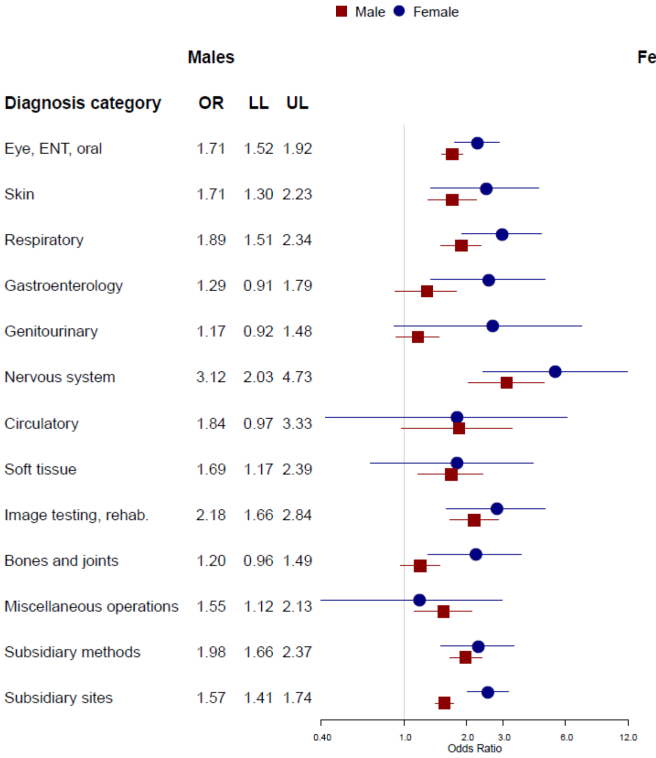

Supplementary figure 7 Attendances to the GP by categories of diagnoses (Read codes), comparing children and young people with vs. without ADHD by age 4-11 vs. 12-17 years (n=48,263)

Reasons for attendance

■ 4 – 11 years ● 12 – 17 years

	4 – 11 years			12 – 17 years		
**Diagnosis category**	**OR**	**LL**	**UL**	**OR**	**LL**	**UL**
Non−specific	4.87	4.29	5.54	7.28	5.75	9.33
Eye, ENT, oral	1.39	1.31	1.47	1.53	1.39	1.68
Skin	1.26	1.19	1.34	1.35	1.22	1.48
Respiratory	1.27	1.20	1.34	1.56	1.42	1.72
Gastroenterology	1.26	1.18	1.35	1.50	1.33	1.70
Musculoskeletal	1.30	1.20	1.40	1.49	1.34	1.64
Injury and external	1.54	1.43	1.66	2.20	1.98	2.44
Mental / behavioural	21.10	19.62	22.70	25.09	22.34	28.18
Genitourinary	1.50	1.39	1.61	1.64	1.44	1.86
Symptoms and signs	1.22	1.11	1.35	1.39	1.13	1.71
Nervous system	2.09	1.90	2.30	1.80	1.54	2.09
Infections	1.19	1.07	1.31	1.79	1.37	2.32
Circulatory	1.41	1.16	1.72	1.31	0.95	1.78
Dyslexia, dyspraxia etc	6.86	5.21	9.07	11.76	6.50	22.16
Endocrine	1.62	1.08	2.39	1.41	0.85	2.25
Neoplams	2.35	1.43	3.78	1.71	0.66	3.97
Congenital malformations	1.96	1.09	3.40	2.96	1.14	7.23

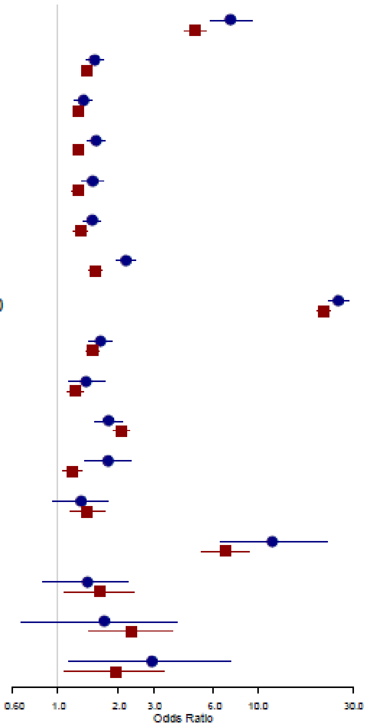

Supplementary figure 8 Attendances to the GP by categories of prescriptions (drug codes), comparing children and young people with vs. without ADHD by age 4-11 vs. 12-17 years (n=48,263)

Reasons for prescription

■ 4 – 11 years ● 12 – 17 years

	4 – 11 years			12 – 17 years		
**Diagnosis category**	**OR**	**LL**	**UL**	**OR**	**LL**	**UL**
Circulatory	3.04	1.81	5.03	1.91	1.14	3.12
Non−specific	0.72	0.28	1.60	4.01	1.77	8.91
Unknown	1.40	1.04	1.87	1.57	0.90	2.63
Nervous system	1.80	1.68	1.93	2.77	2.46	3.11
Endocrine	1.93	1.57	2.37	2.12	1.54	2.90
Anaesthesia	1.89	1.60	2.23	2.18	1.66	2.84
Obstetrics, gynaecological etc.	1.60	1.38	1.84	1.71	1.42	2.07
Dressings	1.47	1.19	1.80	1.93	1.45	2.56
Nutrition and blood	1.60	1.39	1.83	1.57	1.20	2.04
Gastroenterology	1.39	1.27	1.52	1.52	1.30	1.78
Respiratory	1.37	1.29	1.45	1.38	1.25	1.52
Musculoskeletal	1.22	1.10	1.35	1.41	1.23	1.63
Infections	1.29	1.22	1.36	1.45	1.32	1.59
Skin	1.25	1.18	1.33	1.44	1.30	1.58
Ear, nose and oropharynx	1.14	1.05	1.25	1.24	1.09	1.42
Eye	1.03	0.94	1.12	1.34	1.16	1.55
Vaccines etc.	0.85	0.72	1.00	0.95	0.76	1.18

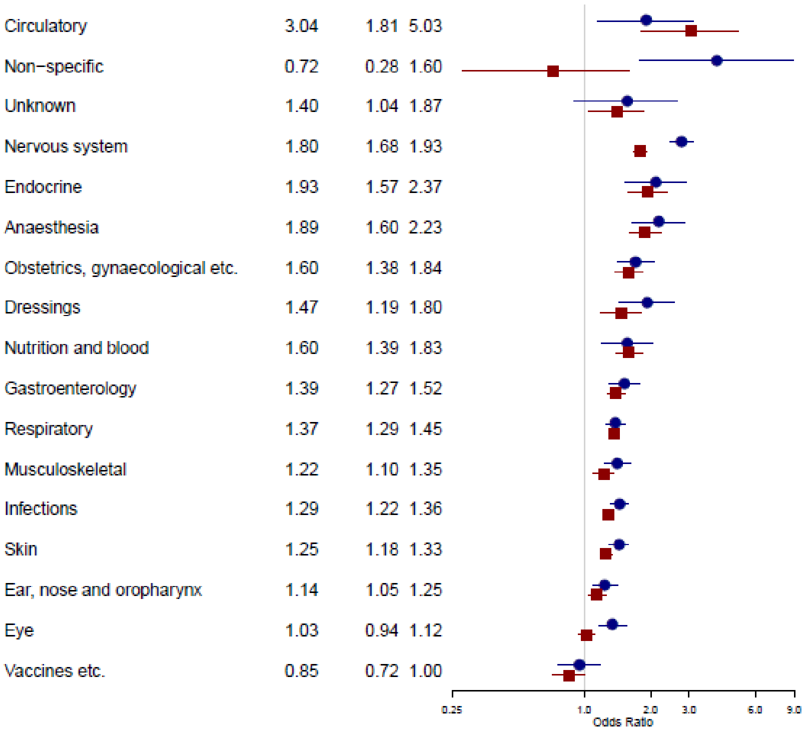

Supplementary figure 9 Medical conditions recorded in the GP medical records, comparing children and young people with and without ADHD by age 4-11 vs. 12-17 years (n=48,263)

Condition

■ 4 – 11 years ● 12 – 17 years

	4 – 11 years			12 – 17 years		
**Diagnosis category**	**OR**	**LL**	**UL**	**OR**	**LL**	**UL**
Epilepsy	4.10	3.22	5.22	4.94	3.39	7.20
Incontinence	2.29	1.97	2.66	2.29	1.54	3.36
Diabetes	1.05	0.54	1.89	1.30	0.57	2.65
Migraines	1.16	0.66	1.93	1.80	1.23	2.59
Language impairment	3.16	2.71	3.69	2.85	1.40	5.60
Learning disability	10.09	7.69	13.31	13.16	7.99	22.34
Sleep disorders	7.12	5.72	8.89	6.61	4.46	9.86
Alcohol abuse	0.71	0.02	5.50	1.76	1.00	2.99
Tic disorders	2.97	2.04	4.29	18.05	8.04	45.73
Asthma	1.32	1.23	1.42	1.39	1.23	1.56
Abdominal pain	1.03	0.92	1.16	1.61	1.36	1.92
Autism	8.32	6.73	10.32	10.11	6.58	15.78
Anxiety	2.09	1.28	3.33	2.41	1.55	3.69
Eczema	1.17	1.05	1.30	1.04	0.83	1.30
Dermatitis	1.17	1.09	1.26	1.17	1.01	1.35
Behaviour codes	21.78	19.75	24.01	33.23	27.36	40.38
Depression	3.71	0.54	21.94	5.77	3.66	9.11
Self−harm	2.48	0.92	6.13	12.71	7.70	21.62
Enuresis	2.42	2.07	2.82	2.21	1.54	3.15

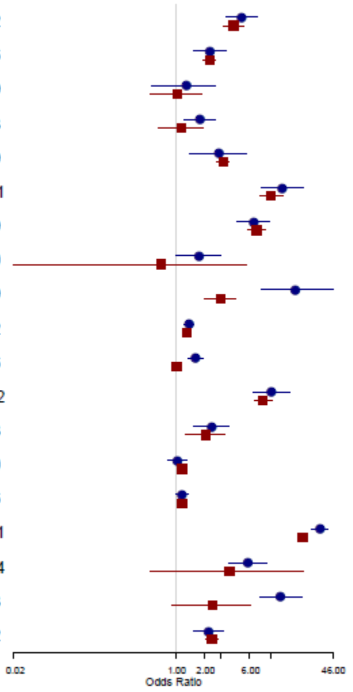

Supplementary figure 10 Admissions to hospital by categories of diagnoses (ICD10 codes), comparing children and young people with vs. without ADHD by age 4-11 vs. 12-17 years (n=48,263)

Reasons for attendance

■ 4 – 11 years ● 12 – 17 years

Diagnosis category	4 – 11 years OR	LL	UL	12 – 17 years OR	LL	UL
Non−specific	1.78	1.51	2.11	3.19	2.47	4.09
Eye, ENT, oral	2.08	1.75	2.46	1.86	1.17	2.90
Skin	1.52	1.07	2.13	2.81	1.65	4.71
Respiratory	1.80	1.57	2.06	1.87	1.39	2.50
Gastroenterology	1.85	1.59	2.15	1.38	1.03	1.83
Musculoskeletal	1.32	0.91	1.86	1.57	0.98	2.45
Injury and external	1.57	1.34	1.84	3.12	2.54	3.82
Mental / behavioural	9.79	7.58	12.72	10.69	7.67	15.02
Genitourinary	1.14	0.87	1.48	1.75	1.09	2.74
Symptoms and signs	1.91	1.65	2.20	2.69	2.12	3.41
Nervous system	3.22	2.46	4.20	4.27	2.65	6.84
Infections	1.37	1.06	1.76	2.00	1.10	3.48
Circulatory	2.18	1.32	3.53	3.29	1.32	7.84
Endocrine	1.02	0.61	1.62	1.60	0.78	3.06
Neoplams	1.42	0.95	2.09	0.69	0.24	1.62
Congenital malformations	1.79	1.41	2.27	1.83	1.08	3.01
External causes	1.47	1.25	1.74	3.25	2.63	4.00

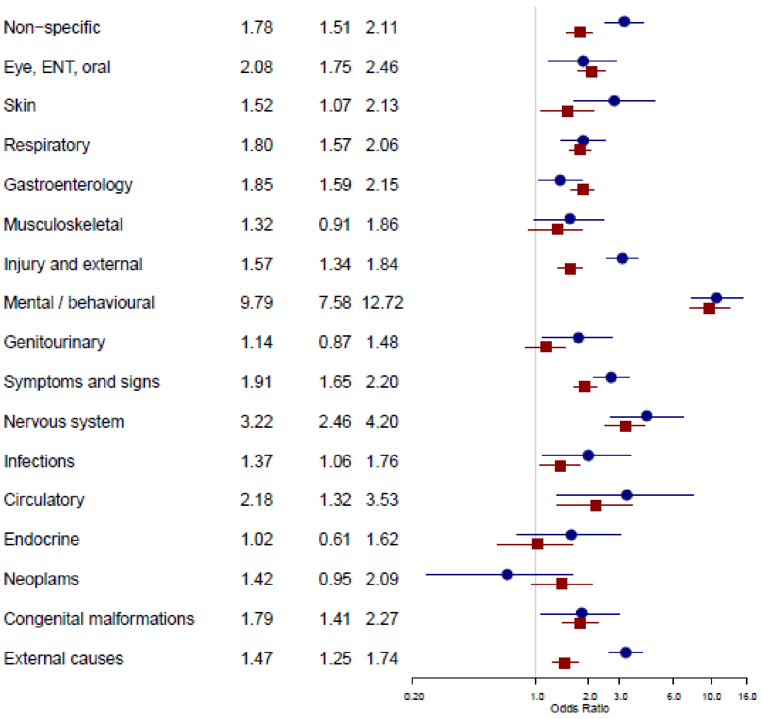

Supplementary figure 11 Admissions to hospital by categories of procedures (OPCS4 codes), comparing children and young people with vs. without ADHD by age 4-11 vs. 12-17 years (n=48,263)

Reasons for operation

■ 4 – 11 years ● 12 – 17 years

Diagnosis category	OR (4 – 11 years)	LL	UL	OR (12 – 17 years)	LL	UL
Eye, ENT, oral	1.88	1.67	2.11	1.45	1.09	1.91
Skin	1.74	1.30	2.33	2.02	1.26	3.16
Respiratory	2.11	1.71	2.58	1.76	0.91	3.25
Gastroenterology	1.43	0.96	2.08	1.57	0.98	2.45
Genitourinary	1.18	0.90	1.53	1.38	0.83	2.23
Nervous system	2.52	1.58	3.95	7.61	3.80	15.70
Circulatory	1.93	1.01	3.52	1.51	0.36	4.91
Soft tissue	1.43	0.96	2.08	2.90	1.49	5.46
Image testing, rehabilitation	2.40	1.82	3.16	1.99	1.20	3.23
Bones and joints	1.00	0.75	1.31	1.89	1.40	2.54
Miscellaneous operations	1.60	1.12	2.24	1.23	0.59	2.36
Subsidiary methods	2.09	1.73	2.53	1.88	1.37	2.55
Subsidiary sites	1.74	1.56	1.94	1.59	1.30	1.94

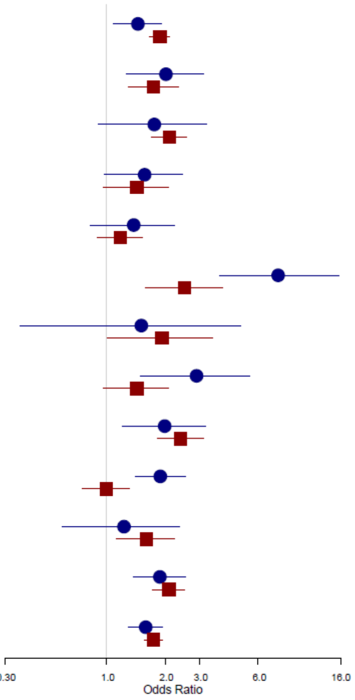

Supplement: Supplementary data [file archdischild-2023-325637supp002.pdf]
